# Neutrophil extracellular traps and interleukin-1β in cystic fibrosis lung disease

**DOI:** 10.3389/fimmu.2025.1595994

**Published:** 2025-07-28

**Authors:** Kayla M. Fantone, Naveen Gokanapudi, Balázs Rada

**Affiliations:** Department of Infectious Diseases, College of Veterinary Medicine, The University of Georgia, Athens, GA, United States

**Keywords:** cystic fibrosis - CF, neutrophil extracellular traps (NET), neutrophil, lung disease, inflammasome, interleukin-1 (IL-1β)

## Abstract

Cystic fibrosis (CF) lung disease manifests through abnormally thick mucus, persistent bacterial infections and a dysregulated innate immune system that involves significant neutrophilic inflammation. Neutrophils, immune cells essential to fight infections, accumulate in large numbers in CF airways and release neutrophil extracellular traps (NETs) into the airway lumen that deliver extracellular DNA, granule content and cytokines including IL-1β. Interleukin-1β, a powerful, proinflammatory cytokine, represents another, significant component of the innate immune system that is dysregulated in CF. Both defense mechanisms become problematic as NETs and IL-1β are present at elevated levels in CF airways, potentially creating a destructive cycle that exacerbates lung damage rather than protects against infections. Therefore, understanding the interplay between IL-1β and NETs is crucial for addressing CF lung disease progression. This review examines the general mechanisms of IL-1β release and NET formation, with particular focus on their role in CF lung disease, and proposes that a self-perpetuating, positive feedback loop between these two innate immune processes represents a major driving force in disease progression. This understanding suggests potential therapeutic targets for interrupting the cycle of inflammation and tissue damage in CF airways.

## 1 Introduction

### 1.1 Cystic fibrosis

Cystic fibrosis (CF) is a genetic disease affecting an estimated 160,000 people worldwide (1, 2). It is caused by mutations in the cystic fibrosis transmembrane conductance regulator (*CFTR*) gene encoded on chromosome 7 (7q31.2) in humans. CF is an autosomal recessive disease that requires two copies of mutated *CFTR* genes to manifest (3). Carriers with a single copy of the mutant *CFTR* gene will pass the mutation to their children but demonstrate no symptoms. An effected individual carrying two mutated copies of *CFTR* can develop CF. One in 30 people are carriers in the USA. Around 2,000 different *CFTR* gene mutations have been identified that are associated with CF (3). The CFTR protein functions as a chloride channel regulating the Na^+^/Cl^-^ balance across mucosal surfaces, especially in the respiratory and digestive tracts. Normal CFTR function facilitates the regular maintenance of mucus levels which is disrupted in CF causing mucus buildup (4–6). CF affects multiple organs that have a secretory function including the digestive and respiratory systems. The absence of a fully functional CFTR anion channel in CF impacts multiple physiological mechanisms indicating its complex and intricate function. Mutant CFTR can lead to -among others- abnormal mucus production in the lung and gastrointestinal system, pancreatic problems, reduced glucose metabolism and impacted circadian rhythm. One of the most impacted organs in CF is the lung and lung disease reduces life expectancy for people with CF (PwCF) (7).

### 1.2 CF lung disease: inflammation and infections

CF airway disease is characterized by chronic inflammation and persistent polymicrobial infections. The defect of the *CFTR* gene alters anion transport across airway epithelial cells which causes dehydration of the airway surface liquid and excessive mucin secretion (7, 8). The excess fluid and mucus in the lungs contribute to airway obstruction and microbial infections leading to chronic inflammation (7, 9). The most abundant inflammatory cells recruited to the CF airway are neutrophils whose main function in the body is to fight off infections (10). The CF lung hosts polymicrobial infections (11, 12). The major pathogens infecting the CF lung are bacteria such as *Staphylococcus aureus*, *Pseudomonas aeruginosa, Haemophilus influenzae, Stenotrophomonas maltophila, Achromobacter* sp*ecies and Burkholderia cepacia* (9). *S. aureus* and *P. aeruginosa* represent the two most prevalent respiratory pathogens in PwCF. *P. aeruginosa* has been observed for a long time as the dominant airway pathogen, infecting 60-75% adult PwCF, but in the past two decades *S. aureus* has become the most common in CF (9, 13, 14). The prevalence of non-tuberculous mycobacterial (NTM) infections has also been rising among PwCF, with *Mycobacterium avium* and *M. abscessus* being the most common ones (11, 12, 15). In addition, PwCF can also be infected with fungal pathogens, such as *Aspergillus fumigatus* (16–18).

**Figure 1:**
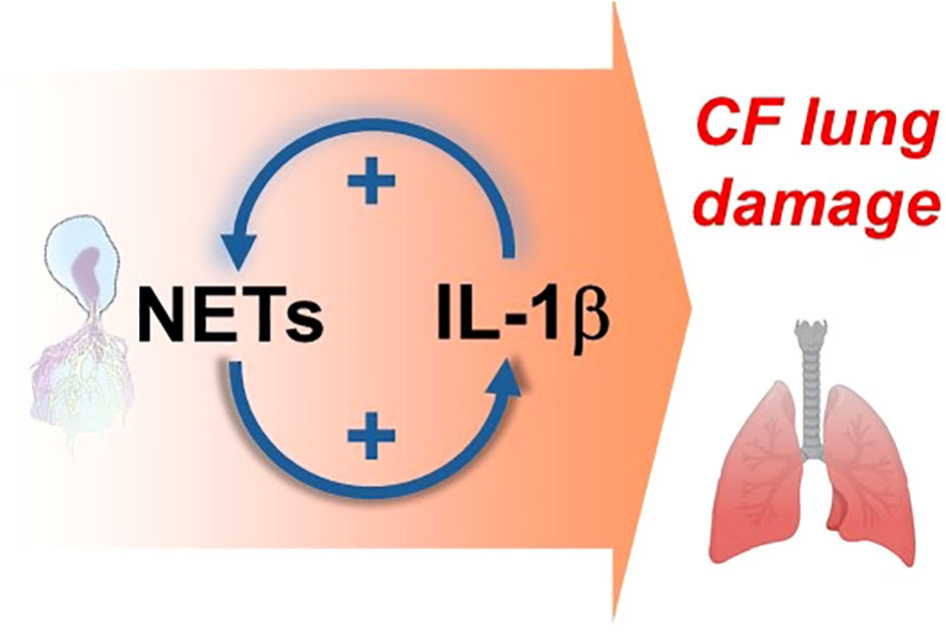
A schematic illustration indicating the hypothesis of this review article that NETs and IL-1β mutually enhance each other and assemble into a positive, innate immune feedback loop that contributes to the chronic inflammation and tissue damage observed in the lungs of PwCF.

Neutrophils are the primary innate cells that respond to *S. aureus* and *P. aeruginosa* challenge in humans (19–22). In CF, neutrophils are recruited to the airways in high numbers but fail to clear a select group of pathogens listed above (23–25). Neutrophils represent a major cause of CF lung damage by releasing their granular and nuclear content driving chronic inflammation (10, 23–25). Proteolytic stress carried out by neutrophil-derived proteases such as neutrophil elastase (NE) and oxidative stress mediated by oxidants produced by neutrophils represent two major mechanisms by which neutrophils directly contribute to lung damage in CF (23, 26). In addition to causing direct tissue damage, neutrophil components released into the CF airway lumen also stimulate the release of cytokines including IL-1β from epithelial and immune cells which attracts additional neutrophils, thereby fueling a feed-forward inflammatory process. Two important components of the innate immune system, and the interplay between them, will be discussed in this article that are hypothesized to be part of a self-perpetuating, proinflammatory process contributing to CF airway disease progression: neutrophil extracellular traps (NETs) and IL-1β (**Figure 1**).

## 2 NETs

### 2.1 NETs: general mechanisms

Neutrophils are abundant innate immune cells equipped with diverse antimicrobial molecules, making them crucial to combat microbial pathogens (27). Neutrophils combat bacteria through 1) intracellular killing by phagocytosis or 2) extracellular killing by forming neutrophil extracellular traps (NETs) or releasing granules by degranulation (27–29). Neutrophils identify pathogens via surface receptors such as pattern recognition receptors, Fc receptors and complement receptors, and engulf them through phagocytosis (27). Once the pathogen is in the phagosome, granules of the neutrophil fuse with the phagosome, and proteases, antimicrobial molecules and oxidants are released into the phagolysosome to create a toxic environment for the engulfed and isolated pathogen (27). Neutrophils also kill pathogens by another mechanism called NET formation (29). During NET formation, neutrophils release their decondensed chromatin decorated with granular content including histones and granule proteins intro the extracellular environment (30). The nucleus of neutrophils loses its lobed morphology in the course of NET formation and chromatin decondenses which is potentiated by enzymes such as NE and myeloperoxidase (MPO) (30). NET formation can be induced by many agonists such as bacterial pathogens and can require the production of reactive oxygen species (ROS) or the activation of different kinases (31). Activation of kinases causes chromatin decondensation leading to histone modifications catalyzed by peptidyl deaminase 4 (PAD4) that converts arginine residues to citrulline (29, 31). In the final stages of NET formation, the nuclear membrane disintegrates, the cell membrane ruptures leading to the release of granular proteins such as NE, citrullinated histones, MPO, defensins and cathelicidins (30, 31).

In addition to the originally described route of NET formation that requires the cell to die (suicidal NETs), another form of NET release has also been documented. In certain cases, neutrophils do not become lysed but only release small amounts of NETs, much faster than suicidal NET formation, remain alive for hours and perform tasks such as migration, ROS production and phagocytosis. This alternative NET pathway has been termed as “viable” NET release and was proposed to mainly involve mitochondrial, not nuclear, DNA (32, 33).

Neutrophils release NETs in response to various stimuli. Different mechanisms of NET formation have been described which include NADPH oxidase (Nox)-dependent and Nox-independent pathways (31). Nox-dependent NET formation is induced by lipopolysaccharide (LPS) found in gram-negative bacteria that binds to TLR4 on the surface of neutrophils and induces ROS production by the NADPH oxidase (Nox2) (31, 34). During Nox-dependent NET release, ROS generated by Nox2 induces the disintegration of granule and nuclear membranes, allowing for NE and MPO to interact with the nucleus to cleave histones and cause chromatin decondesation (30, 31, 34). On the other hand, calcium ionophores can also induce NET formation in a Nox-independent manner (35). PAD4 is abundantly present in the cytosol of neutrophils, can bind to calcium and translocate into the nucleus (31, 36). The PAD4 enzyme deiminates histone arginine residues into neutral citrulline amino acids causing chromatin decondensation (24, 31). There is further evidence to suggest that calcium-activated potassium channel of small conductance (SK3 channel) and mitochondrial ROS (mROS) are also required to induce Nox-independent NET formation (37). Calcium ionophores stimulate mROS through influx via the SK3 channel to induce Nox-independent NET extrusion (37). Another important step underlying NET formation is transcriptional firing at promoter regions that help to mediate DNA decondensation (31, 38). It has been found that transcription of Erk-, Akt-, p38- and cSrc-regulated genes are the primary drivers of Nox-dependent NET formation, whereas transcription of Akt-, p38-, cSrc-, PyK2- and Jnk-regulated genes drive Nox-independent production of NETs (31, 38). Lastly, histone modifications are also important components for NET formation in neutrophils. Histone acetylation plays a relevant role in NET formation and causes neutralization of the positive charges on the histones that promotes chromatin decondensation (31, 39).

Dysregulation of NET formation and/or NET clearance have been associated with the severity of several lung diseases such as CF, acute respiratory distress syndrome, acute lung injury and airway infections including COVID-19 (40, 41). COVID-19 caused by the respiratory virus SARS-COV-2 is characterized by severe lung disease that requires hospitalization in some patients (42). In the NETCOV2 study, the number of days with severe hypoxemia in the intensive care unit patients with SARS-CoV-2-related pneumonia correlated negatively with blood NET levels measured at 1-day post-admission (42). The absence of decrease of the blood NET levels between day-1 and day-3 discriminated patients who died within days (42). This and other studies strongly suggest a pathologic role of NETs in severe SARS-CoV-2-associated pneumonia (43, 44). A pathologic role of exaggerated neutrophil recruitment and NET release in lung function decline, and a prognostic role of the blood neutrophil:lymphocyte ratio for future disease severity have been indicated in severe COVID-19 (45–47). NETs have been proposed as the delivery platform to bring the tissue-damaging intracellular cargo of neutrophils to the airway lumen including reactive oxidants leading to lung tissue damage in severe COVID-19 (48). Overall, while NETs play an important role in innate host defense by trapping extracellular pathogens, they have been proposed to contribute to the pathologies of several illnesses including CF lung disease.

### 2.2 NETs in CF

**Figure 2:**
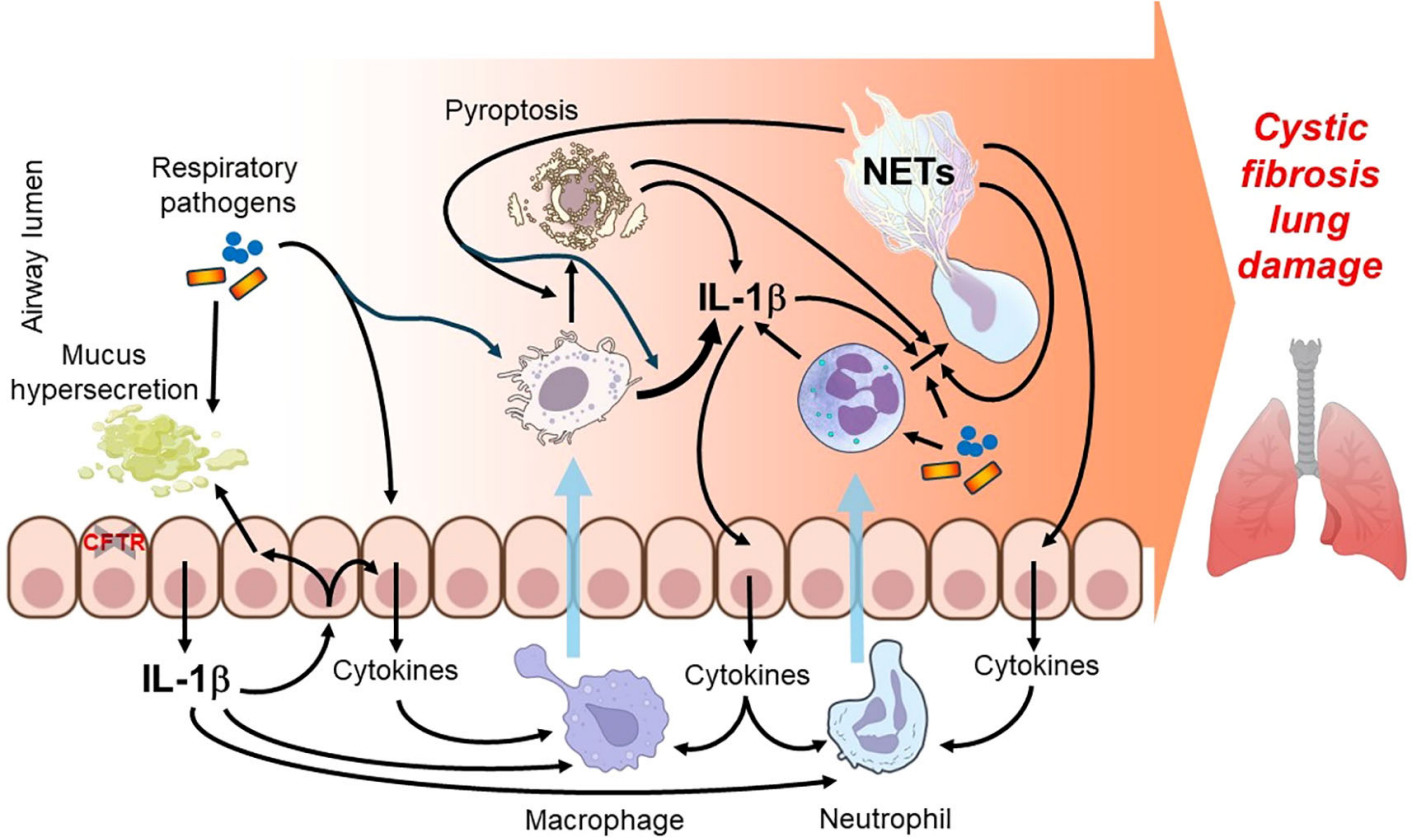
Proposed mechanisms connecting NETs, IL-1β and other components of the airways that support chronic inflammation in CF. CFTR deficiency in airway epithelial cells leads to excessive release of mucus and cytokines including IL-1β. Produced cytokines recruit innate immune cells, macrophages and neutrophils. Mucus overproduction and impaired mucociliary clearance attract bacterial pathogens that stimulate the recruited immune cells and the airway epithelium to generate cytokines and to recruit additional leukocytes, leading to a self-perpetuating inflammatory process. In addition, macrophages produce IL-1β and undergo pyroptosis while neutrophils release NETs, which overall deliver the intracellular content of these innate cells to the airway lumen. NETs activate macrophages yielding IL-1β release and pyroptosis, while microbial and host molecules, likely including IL-1β, induce further NET extrusion. The complex network of the proposed innate immune mechanisms indicated by the arrows fuel chronic inflammation and lung disease in CF in a positive feed-forward fashion. Prepared with BioRender.

In CF, chronic inflammation in the airways is characterized by a massive neutrophil influx with subsequent release of NETs (34) (**Figure 2**). All these changes lead to persistent lung injury through the accumulation of dysfunctional neutrophils releasing their DNA and cytotoxic antimicrobial content such as MPO, lysozyme, lactoferrin, NE, defensins, gelatinase, cathelicidins and cathepsins. The CF sputum and bronchioalveolar lavage fluid contain high levels of neutrophil granular components whose concentrations correlate with the severity of lung disease in CF (10, 49–51). The massive influx of neutrophils and the release of neutrophil DNA in the bronchioles in the CF lung aggravate mucus viscosity providing a suitable environment for the colonization of infectious bacteria (10, 49–51). The excessive mucus production in CF airways not only contributes to the establishment and persistence of bacterial infections but also to the clogging of the airways resulting in tissue damage and subsequent disease pathology. *S. aureus* and *P. aeruginosa* are strong NET inducers (52–58) (**Figure 2**). DNA released from neutrophils via NETs has been proposed to promote bacterial colonization and biofilm formation in the CF airways (59, 60). CF neutrophils were shown to have more robust, spontaneous NET formation than non-CF control cells due to their delayed apoptotic response and longer survival (61–63). DNA in the CF sputum demonstrates characteristics of NETs and NET levels correlate with lung disease in CF (50, 64).

PwCF develop autoantibodies that target neutrophil or NET components, such as bactericidal permeability-increasing protein, carbamylated proteins, PAD4 and DNA (65–69). Blood levels of anti-PAD4 antibodies and anti-double-stranded DNA IgA autoantibodies are elevated in CF and associate with worsened lung function (67, 68). The reported autoantibody pattern in CF is different from that described in well-established and characterized autoimmune diseases, such as rheumatoid arthritis or systemic lupus erythematosus (SLE) (65, 67). Not only IgG but also IgA autoantibodies can be strong stimulants of neutrophils (70, 71). These autoantibodies could represent additional stimuli for neutrophils to release NETs and to fuel NET-mediated inflammation in CF (65, 67).

Overall, NETs have been documented in the CF airways in abundance, correlate with lung function decline, are not capable of clearing bacterial infections, and likely provide a platform for delivering the proinflammatory and tissue-damaging neutrophil intracellular content to the airway lumen to fuel chronic inflammation (**Figures 1**, **2**).

## 3 Interleukin-1β

### 3.1 IL-1β in CF

Progressive lung disease in CF results in excess mucus in the airways and trapping of bacteria, leading to infections, inflammation, and overall respiratory failure. Release of NETs and microbial molecules originating from bacterial infections activate a multimeric protein complex known as the inflammasome, which is present in many innate immune cell types including neutrophils and macrophages (72). Macrophages infiltrate the CF airways to eliminate neutrophil debris and help fight microbial pathogens, contributing to chronic inflammation (**Figure 2**).

**Figure 3:**
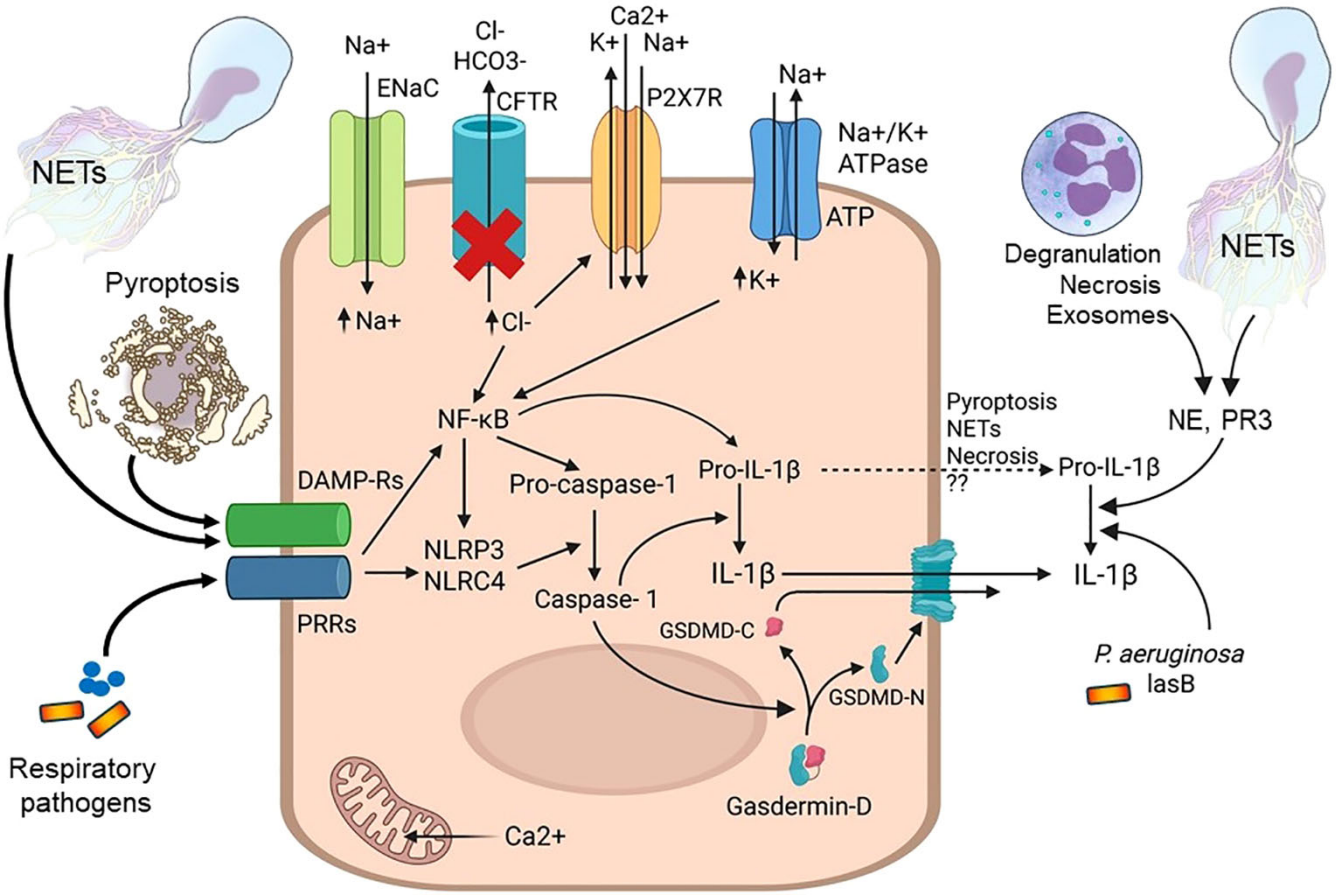
Proposed molecular mechanisms driving the generation of bioactive IL-1β in innate immune cells in CF airways. Innate immune cells in the CF lung are stimulated by microbial molecules derived from respiratory pathogens via patter recognition receptors (PRRs) or by host molecules originating from neutrophils that had undergone NET formation or pyroptosed macrophages via receptors recognizing danger-associated molecular patterns (DAMP-R). DAMP-Rs and PRRs activate the transcription factor NF-κB that up-regulates the expression of the genes encoding inflammasome components, pro-IL-1β and pro-caspase-1. Ion channels and transporters localized to the plasma membrane can influence signaling pathways as indicated. Inflammasomes (NLRP3, NLRC4) cleave pro-caspase-1 into active caspase-1. Caspase-1 cleaves pro-IL-1β into bioactive IL-1β and gasdermin-D into two fragments, the secreted component, gasdermin-C (GSDMD-C) and the pore-forming unit, gasdermin-N (GSDMD-N). IL-1β leaves the cell via the gasdermin pore. When innate immune cells die by pyroptosis, necrosis or NET formation, pro-IL-1β has been proposed be released into the airway lumen. Extracellular pro-IL-1β can be cleaved by neutrophil proteases (neutrophil elastase, NE; proteinase 3, PR3) released from neutrophils via the indicated, different mechanisms or by the lasB protease derived from *P. aeruginosa*. The red cross indicates CFTR deficiency. Prepared with BioRender.

IL-1β serves as a key inflammatory mediator in the airway, and its secretion involves inflammasome-mediated processing (72). In PwCF, IL-1β was detected in the bronchoalveolar lavage fluid even without infection, its airway levels increased in the presence of bacterial infection and correlated with the neutrophil count and NE activity in CF airways (73). These data and others suggest that IL-1β plays an important role in recruiting and/or stimulating neutrophils in CF. Indeed, IL-1β was shown to recruit neutrophils to the airways in sterile lung injury and airway infection models in mice (74). It is unclear whether PwCF have increased IL-1β production in the absence of infection due to an intrinsic increase in NF-κB activity or because of the loss of CFTR function (75). However, without a functional CFTR, ENaC channels are dysregulated leading to increased intracellular Na^+^ levels and increased efflux of K^+^ (76). Upregulation of K^+^ efflux in the CF airways is an extracellular stimulus for NLRP3 inflammasome activation, leading to subsequent release of IL-1β and IL-18. The combination of K^+^ efflux and microbial stimuli such as pathogen-associated molecular patterns (PAMP), lead to excessive NLRP3 inflammasome activation and downstream proinflammatory cytokine release (**Figure 3**). *P. aeruginosa* and *S. aureus* lung infections increase airway levels of IL-1β in mice (77, 78). Levels of IL-1β in the lung are also higher in CF mice infected with either *P. aeruginosa* or *Aspergillus fumigatus*, a fungal CF respiratory pathogen (79). Anakinra, a recombinant non-glycosylated homolog of the human IL-1 receptor antagonist (IL-1Ra), protects CF mice from infections and NLRP3-mediated inflammation following either *P. aeruginosa* or *A. fumigatus* infection (79).

One study found that blocking ENaC channels in human bronchial epithelial cells with a naturally occurring 18-residue peptide restores Na^+^ and K^+^ levels, reduces NLRP3-mediated IL-1β and IL-18 release and attenuates inflammation (76). Sterile inflammation in PwCF displays IL-1β-induced expression of secreted airway mucins in primary human bronchial epithelial cells, which can be prevented by IL-1Ra treatment (80–82). Evidence suggests an important role of the IL-1 signaling pathway early in CF lung disease by contributing to neutrophilic airway inflammation and mucus hypersecretion, in the absence of any detectable infection (83). IL-1β may be the dominant cytokine in the inflammatory environment of the CF lung, especially after the onset of bacterial infections (84) (**Figure 2**). The IL-1R is predominantly expressed on cells that also produce IL-1β: innate immune cells including neutrophils and macrophages, and airway epithelial cells; suggesting a positive feedback loop for production and release of IL-1β in the CF airways (84). Airway epithelial cells undergoing necrosis release IL-1β through activation of the IL-1R-MyD88 signaling pathway that is known to induce neutrophilic inflammation in mice with CF-like lung disease (85). The inflammasome has been demonstrated to be important in the pathogenesis of lung fibrosis and studies suggest its dependence on the IL-1R pathway (85). PwCF may also have a predisposition to dysregulated IL-1β signaling due to genetic polymorphisms (86). IL-1β polymorphisms are present among PwCF and specific single nucleotide polymorphism (SNP) in the IL-1β gene have been associated with lung disease in CF. IL-1β levels are enhanced in PwCF harboring these SNPs compared to patients without them, and IL-1β-mediated inflammation is further increased in the presence of respiratory infection (86, 87).

On a per cell basis, macrophages represent the major source of IL-1β, while neutrophils and airway epithelial cells also release this cytokine at lower levels per cell (88–90). Considering their larger cell number in the CF airways, neutrophils and airway epithelial cells also represent significant sources of IL-1β (**Figure 2**). *P. aeruginosa* stimulates IL-1β release *in vitro* in macrophages (91, 92), neutrophils (93) and airway epithelial cells (90, 94). *S. aureus* also induces IL-1β synthesis in innate immune cells (95–97) *in vitro*.

CF airway epithelial cells demonstrated significantly higher levels of released IL-8 and IL-6 in response to IL-1β stimulation, in the absence of any infection or microbial stimuli *in vitro* (98–100). This proinflammatory phenotype of CF airway epithelial cells is likely due to a combination of different factors including 1) endoplasmic reticulum stress that develops because of the intracellular accumulation of misfolded CFTR proteins in case of certain CFTR mutations, impaired regulation of 2) ion transport and 3) intracellular signaling pathways (101, 102). Macrophages isolated from PwCF have also been reported to release larger amounts of cytokines including IL-1β when stimulated with LPS *in vitro* (103). These data indicate that endogenous CFTR deficiency can push airway epithelial cells and macrophages towards a “proinflammatory” phenotype that manifests -among other things- in heightened proinflammatory cytokine release.

Overall, several mechanisms are likely responsible for the increased IL-1β levels observed in the CF lung.

### 3.2 Inflammasomes

The inflammasome provides a protective innate immune mechanism that activates caspase-1 and leads to the proteolytic cleavage of pro-IL-1β and pro-IL-18 to induce inflammation in response to PAMPs and danger-associated molecular patterns (DAMPs) (104) (**Figure 2**). Toll-like receptors (TLRs) are the key inducers of the transcription of proinflammatory cytokines, IL-1β and IL-18, through NF-κB stimulation (**Figure 3**). Nod-like receptor (NLR) family protein members contain a nucleotide binding domain (NBD), a variable N-terminal domain [either pyrin domain (PYD) or the caspase recruitment domain (CARD)], and a C-terminal leucin-rich repeat (LRR) region (105, 106). The assembly of the inflammasome requires interaction between the CARD-CARD or PYD-PYD domains. The CARD domain assembles with the pyrin domain to recruit caspase-1 through the adaptor molecule apoptosis-associated speck-like protein containing a CARD (ASC) (107). Two inflammasomes have been under investigation in CF lung inflammation in greater detail: nucleotide-binding oligomerization domain-like receptor pyrin domain-containing protein 3 (NLRP3) and NLR family CARD domain-containing protein 4 (NLRC4) (79). NLRC4 has been more extensively studied in CF than NLRP3, however, NLRP3 has been investigated in general in more detail (79, 108). The NLRP3 inflammasome is comprised of a N-terminal PYD domain, a central binding domain and a C-terminal LRR domain (105, 106, 109). The CARD-domain in the inflammasome contains the NLRs which assemble with the pyrin domain to recruit caspase-1 through the adaptor molecule, ASC (105, 106, 109). NLRs are named according to their domain structure and contain a nucleotide-binding domain (NBD), a signaling domain (CARD or PYRIN) and a leucine-rich repeat (LRR) domain that mediates ligand binding (110). Two signals are required for the NRLP3 inflammasome to produce bioactive IL-1β and IL-18: priming and activation. Priming of the inflammasome is the first signal and it manifests in the transcription of inflammasome proteins, pro-IL-1β and pro-IL-18 themselves. This priming step is typically initiated by TLR or cytokine receptor activation via NF-κB-dependent transcriptional upregulation of the affected genes. Activation of the inflammasome is the secondary signal resulting in the proteolytic cleavage of its target protein, pro-caspase-1, into enzymatically active caspase-1, that will then next cleave pro-IL-1β and pro-IL-18 into their bioactive, final forms (**Figure 3**). The NLRP3 inflammasome can be activated by a wide variety of signals ranging from bacterial toxins, pore-forming complexes, extracellular ATP, microcrystals and stimuli leading to lysosome rupture to release calcium, potassium ions and ROS (105, 106, 109, 111) (**Figure 3**). Accordingly, the activation of the NLRP3 inflammasome is triggered by an abundance of receptors and particles during infections and sterile inflammation. Caspase-1 also processes cytosolic gasdermin-D to unleash its pore-forming, N-terminal domain, resulting in the release of mature cytokines, alarmins and pyroptotic cell lysis (111) (**Figure 3**).

While the NLRP3 inflammasome is an essential part of the innate immune system, its overactivation can result in enhanced inflammation, tissue damage and contribute to disease pathologies in inflammatory conditions (112). Therefore, understanding the precise regulation of NLRP3 inflammasome activation is critical for fighting infections and curbing inflammation (113). Although cleavage of IL-18 and IL-1β takes place in the cytoplasm, IL-1β, in particular, has more than one pathway to exit the cell such as via exocytosis in secretory lysosomes, shedding of the plasma membrane in microvesicles, direct release by exosomes or through pyroptosis (114–116). Pyroptosis is a form of cell death dependent on caspase-1 and initiated by bacteria and a member of the NLR family receptors (117) (**Figures 2**, **3**). IL-1β is involved in a variety of cellular activities such as cell proliferation, differentiation and apoptosis (113). IL-1β may also be processed independently of caspase-1 suggesting a larger role in the inflammatory responses (113). In summary, IL-1β has been detected at elevated levels in CF airways and thought to be a significant factor in driving chronic lung inflammation (**Figures 1**–**3**).

### 3.3 Inflammasome-dependent IL-1β release in CF

A line of several, *in vitro* and *in vivo*, studies suggest a role of inflammasome-mediated IL-1β activation in lung diseases including CF. Mice deficient in *Asc* have decreased IL-1β levels and are protected against lung fibrosis (118). IL-1Ra inhibits inflammasome activation in human CF bronchial epithelial cells yielding reduced IL-1β production (79). Anakinra, an IL-1R antagonist, displayed protective effects on neutrophilic inflammation in a Scn1b-Tg mice model of CF lung disease (85). Anakinra has also been shown to reduce NLRP3-mediated inflammation in *Cftr*-deficient mice and in human CF bronchial epithelial cells (79). Anakinra also regulates mROS production that is required for downstream activation of the NLRP3 inflammasome (118). NLRP3 depletion leads to robust reduction of IL-1β production in mice and human bronchial epithelial cells (79). Therefore, NLRP3 is likely the most significant mediator of IL-1β generation and release in the CF lung.

Neutrophils and macrophages not only respond to IL-1β but also produce bioactive IL-1β themselves. These cell types express more than one inflammasome type and therefore multiple inflammasomes may play a role in their IL-1β secretion. Macrophages secrete proinflammatory cytokines that enhance neutrophil responses, and vice versa, neutrophils release cytokines that increase the responsiveness of macrophages (**Figures 1**, **2**). It is thought that macrophage dysfunction contributes to the early cascade of inflammatory events leading to chronic infection and inflammation in CF (119). CF macrophages demonstrate enhanced IL-1β secretion and reduced surface expression of TLR5 leading to the diminished bacterial phagocytosis and promotion of chronic inflammation (119). The excessive inflammatory response of CF macrophages also likely affects CF neutrophils contributing to a cascade of inflammatory signals originating from both cell types in CF.

### 3.4 Inflammasome-independent IL-1β synthesis in CF

While IL-1β is typically formed as a result of inflammasome activation, there is evidence that IL-1β can be generated independently of any inflammasome. Neutrophil-derived proteases, for instance, have been proposed to cause proteolytic cleavage of extracellular pro-IL-1β to bioactive IL-1β, contributing to the sustained inflammatory airway environment in CF (120) (**Figure 3**). Specifically, proteinase 3 (PR3) that colocalizes with NE in neutrophil primary granules, enhances bioactive IL-1β secretion (121–123). Neutrophil serine proteases have been shown to cleave IL-1β in several murine models of sterile inflammation (122–124). Inhibition of NE activity in *Nlrp3*-deficient mice infected with *S. aureus* significantly reduced airway levels of IL-1β, suggesting that NE-mediated conversion of IL-1β takes place *in vivo* (77). Furthermore, it has been shown that IL-1β is detectable in the lavage fluid of CF children (mean range of 3.8 years of age) in the absence of any infection, which is associated with enhanced NE activity and worsened structural lung damage (87). In a corneal, not pulmonary, *P. aeruginosa* infection mouse model, most of IL-1β was generated by NE, not capsase-1 or inflammasomes (125). Therefore, neutrophil-derived and NE-cleaved IL-1β could be a significant contributor to the total IL-1β released into the CF airway (**Figure 2**).

In addition, the *P. aeruginosa* protease lasB has also been shown to cleave IL-1β *in vitro* and to contribute to airway inflammation in a *P. aeruginosa* lung infection model in mice (126, 127). These findings suggest that not only host (inflammasome-dependent and -independent), but microbial proteases can also contribute to the generation of bioactive IL-1β in the CF airways and to ongoing inflammation (**Figure 3**).

### 3.5 Pathogen-independent activation of the NLRP3 inflammasome

NLRP3 inflammasome activation provides a vital element of host defense against invading pathogens in the lung. NLRP3 is, however, also activated in response to signals of cellular stress, independent of microbial stimuli. CFTR dysfunction leads to impaired endosomal trafficking, cytoskeleton disassembly and inflammasome activation through NF-κB to produce bioactive IL-1β in the CF airway epithelium (128). *CFTR* deficiency also results in the accumulation of ceramide that activates the NLPR3 inflammasome (129). βENaC mice harboring mucus-obstructed bronchioles characteristic of CF lung disease presented enhanced NLRP3 inflammasome activation in their epithelial cells and infiltrating leukocytes and a decrease of intracellular sphingosine-1 phosphate (S1P) signaling (130). Ceramide is the central sphingolipid metabolite that precedes S1P production to induce inflammatory responses (131, 132). S1P and ceramide signaling are part of the sphingolipid rheostat regulatory system with opposing effects (132). It is not fully understood how ceramide activates the NLRP3 inflammasome, but studies suggest that intracellular fatty acid crystals may recruit ASC and trigger ROS release (129, 133, 134). Pretreatment of airway epithelial cells of the βENaC mice with NLRP3 inflammasome inhibitors reduced their exaggerated inflammatory response (76). CF monocytes display enhanced NLRP3 inflammasome activity with increased IL-18, IL-1β, caspase-1 and ASC specks (76). In addition, treatment of CF monocytes with CFTR modulators, ivacaftor and tezacaftor, shows a reduction in IL-18, IL-1β, caspase-1 and ASC specks (135). Levels of IL-18 and IL-1β are also reduced in the serum of PwCF following treatment with CFTR modulators (135). The CFTR modulators partially restore CFTR function and alter CFTR-ENaC coupling to reduce the elevated amiloride-sensitive Na^+^ transport (136, 137). CFTR dysfunction also influences Ca^2+^ influx promoting a proinflammatory response in mitochondria (138) (**Figure 3**). Ca^2+^ influx promotes NLRP3 inflammasome activation (138). In human macrophages, Ca^2+^ is essential for the release of IL-1β and NLRP3 inflammasome activation (139) (**Figure 3**). Also, CF airway neutrophils exposed to LPS demonstrate increased cytoplasmic levels of the M2 isoform of pyruvate kinase that is known to increase pro-IL-1β synthesis while affecting the mitochondria to shuffle glucose between the tricarboxylic acid cycle to glycolysis (140). This results in increased glycolysis in neutrophils mitochondria and enhanced mROS generation, which is a major activator of the NLRP3 inflammasome (140). The signaling pathways for activation of the NLRP3 inflammasome in CF are not well-understood and require further investigations.

### 3.6 CF pathogen-dependent activation of the NLRP3 inflammasome

While the NLPR3 inflammasome can be activated by a broad range of different, non-microbial stimuli described above, bacterial pathogens and their PAMPs are also its strong activators. Pathogens in the lung trigger NLRP3 signaling directly through PAMPs and indirectly via host-stress signals through DAMPs. Pathogens can affect host cells by inducing K^+^ efflux, stimulating ROS production, Ca^2+^ mobilization, mitochondrial destabilization or lysosome rupture, all of which act as DAMPs for the innate immune system (**Figure 3**). *P. aeruginosa* is known to trigger the NLRC4 inflammasome in response to its flagellin and type 3 secretion system, however, *P. aeruginosa* loses those virulence factors over time to evade immune recognition and to develop chronic infection (141, 142). *P. aeruginosa* isolates from PwCF failed to induce inflammasome activation, IL-1β release and pyroptotic cell death in primary macrophages isolated during both stable infection and exacerbation (141). This was attributed to diminished expression of inflammasome ligands and reduced bacterial motility (141). CF human bronchial epithelial cells infected with a *P. aeruginosa* reference strain displayed mitochondrial perturbation to trigger NLRP3 activation, IL-1β and IL-18 processing (138). The flagellin was shown to be the responsible inducer for the mitochondrial Ca^2+^ uniporter to signal enhanced NLRP3 activation (138). Therefore, one of the reasons why *P. aeruginosa* loses its flagellum overtime is thought to prevent NLRP3 inflammasome activation while another important reason is to avoid recognition by neutrophils and to evade neutrophil-mediated killing (52). Clinical isolates of *P. aeruginosa* from PwCF collected at the beginning of infection induce inflammasome signaling, cell death and expression of IL-1β in macrophages, however, chronic isolates displayed poor inflammasome activation and proinflammatory cytokine release (143). Also, genetic polymorphisms in NLRP3 have been linked to higher rates of *P. aeruginosa* colonization in CF macrophages resulting in worsened lung function overtime (144).

As the currently most predominant respiratory pathogen in CF, *S. aureus* is also known to activate the NLRP3 inflammasome. A pore forming toxin of *S. aureus*, the Panton-Valentine Leukocidin (PVL), induces NLRP3 inflammasome in human primary monocytes, macrophages, and neutrophils (145). This suggests that toxins released by this pathogen may trigger inflammasome signaling, however, it remains unclear whether *S. aureus* CF clinical isolates produce these toxins (145). It is believed that PVL is the predominant factor of *S. aureus* to trigger inflammasome activation in human phagocytic cells to facilitate inflammation in the lung (145, 146). IL-1β released by PVL-intoxicated macrophages causes secretion of IL-8 and monocyte chemotactic protein-1 to recruit neutrophils (146). NLRP3 inflammasome activation in CF macrophages and neutrophils infected with bacterial clinical isolates have yet to be characterized, therefore, it remains largely unknown how chronic infection in PwCF affects inflammasome-mediated inflammation.

## 4 Interplay between NETs and IL-1β

### 4.1 NETs and IL-1β in disease pathologies

NETs and IL-1β have been studied in CF separately but their potential interactions remain largely unexplored. This work proposes that NETs and IL-1β mutually enhance each other’s generation and are therefore parts of a positive feedback loop that is a significant force in the chronic inflammatory process in the CF lung.

Neutrophils play a primary role in inflammation in many autoimmune and chronic inflammatory diseases such as adult-onset Still’s disease (AOSD), SLE, rheumatoid arthritis, gout, asthma and COPD (147–150). In several autoimmune diseases it has been shown that NETs trigger enhanced activation of NLRP3 expression in macrophages (151–153). In rheumatoid arthritis, treatment with an NLRP3 inhibitor improved inflammation indicating a crucial role of IL-1β in disease pathogenesis (152). It remains, however, largely unknown how NETs trigger NLRP3 inflammasome activation in rheumatoid arthritis. In AOSD, galectin-3, a protein known for macrophage activation, was shown to promote ASC and NLRP3 association and inflammasome activation (151). Lupus macrophages have increased inflammasome activation and IL-1β release in response to NETs and the NET-associated protein LL-37 (153). NETs have been described to play a central role in activating the NLRP3 inflammasome in macrophages via LL-37 triggering K^+^ efflux from the cell via the purinergic receptor P2X7R (154) (**Figure 3**). A cooperation between NETs and IL-1β has also been proposed to be behind the pathologies in conditions such as venous thrombosis (155), acute respiratory distress syndrome/acute lung injury (156) and cancer (157, 158).

The sputum of neutrophilic asthma and COPD patients contain elevated levels of extracellular DNA and increased gene expressions of NLRP3 and IL-1β, which correlate with the severity of lung disease (159). The major difference between these autoimmune diseases and CF is that chronic microbial infections are abundant in CF. More recently, in severe cases of COVID-19, IL-1β and NETs were proposed to lead to excessive alveolar and endothelial damage, suggesting a feed-forward loop involving both mechanisms (160). It is believed that the increased levels of IL-1β during SARS-CoV-2 infection activate more neutrophils resulting in increased NET extrusion, which in turn enhances clot formation, endothelial and alveolar damage in the lung (160).

### 4.2 The potential role of NETs in IL-1β production in CF

NETs can trigger macrophages and monocytes to release IL-1β through NLRP3 inflammasome activation causing persistent proinflammatory signals (**Figures 2**, **3**). NETs were shown *in vitro* to promote the activation of the NLRP3 inflammasome and pyroptosis in peripheral blood mononuclear, pulmonary microvascular endothelial cells and keratinocytes (161–163).

In CF, NLRP3 has been found to be the dominant inflammasome, mediating IL-1β release in neutrophils (164). Intracellular accumulation of Cl^-^ in PwCF stimulates the secretion of IL-1β, acting as an autocrine positive feedback loop by regulating NLRP3 and caspase-1 activation (165). P2X7R promotes inflammasome activation in monocytes/macrophages (166). P2X7R is overexpressed in CF monocytes and its inhibition decreases NLRP3 expression and IL-1β release (167). The NLRP3 inflammasome results in damaging levels of inflammation that may be beneficial to the pathogens surviving in the airways of PwCF. NETs are present in large amounts in the CF lung; however, pathogens are able to evade being killed by NETs, therefore, NETs may contribute to the enhanced NLRP3 activation observed in CF. Although chronic *P. aeruginosa* infection has been shown to evade NLRP3 activation, type 3 secretion system-negative *P. aeruginosa* is detected by guanylate binding protein 2 and interferon-inducible protein which causes bacterial lysis (168). The bacterial lysis activates caspase-11 which inhibits proliferation of bacteria and activates the NLRP3 inflammasome (168). On the other hand, Type III interferon has been shown to contribute to the pathogenesis of *S. aureus* infection in the airway, influencing the NLRP3 inflammasome and associated proinflammatory cytokine production (77). It was also demonstrated that IL-1β production during early infection was dependent on caspase-1, whereas during chronic infection, IL-1β generation was dependent on NE (77). Type III IFN activates inflammasome signaling through the JAK/STAT pathway (77, 169). NE and caspase-1 are required for IL-1β processing in response to *S. aureus* lung infection and the inhibition of both, NE and NLRP3, reduce airway levels of IL-1β and clears bacterial airway infection (77).

Lethal NET formation also represents a process by which the intracellular neutrophil content is released into the extracellular environment, and NETs have also been proposed as a platform for extracellular delivery of pro-IL-1β, the inactive preform of IL-1β (170) (**Figure 3**). Pyroptosis, necrosis, exosome release or breakdown following apoptosis of immune cells have all been proposed as alternative mechanisms for the extracellular delivery of pro-IL-1β (170, 171). Once in the extracellular space, pro-IL-1β can be cleaved into bioactive IL-1β by neutrophil-derived (NE, PR3) or bacterial proteases (*P. aeruginosa* lasB) (**Figure 3**) (121–123, 126, 127).

In summary, the NLRP3 inflammasome is activated by the innate immune system to aid in recruitment of immune cells for bacterial clearance, however, in the CF lung this may be detrimental by feeding lung tissue damage and chronic infections.

### 4.3 The potential role of IL-1β in NET formation in CF

While it has been studied more exhaustedly how NETs affect inflammasome activation, interestingly, much less information is available about how IL-1β interferes with NET release. The best this topic has been studied is in the context of gout, an autoinflammatory condition characterized by monosodium urate (MSU) crystal-induced inflammasome activation and IL-1β release in macrophages, followed by neutrophil recruitment and NET extrusion (172). Neutrophils play a major role in mediating inflammation in gout, and IL-1β is a crucial cytokine being released in large quantities from macrophages activated by MSU crystals (173). While IL-1β itself does not stimulate NETs *in vitro*, it was shown to significantly enhance MSU crystal-induced NET release in neutrophils, suggesting that the two innate immune mechanisms could promote each other in a positive feed-forward manner (173). Anakinra inhibited the potentiation of MSU crystal-stimulated NET formation by IL-1β *in vitro* (173, 174). It remains an interesting question whether IL-1β also promotes NET formation induced by stimuli other than MSU crystals that are clinically relevant for CF lung disease such as *P. aeruginosa, S. aureus*, other CF pathogens or microbial or host molecules.

## 5 Clinical considerations

The dysregulated and overactive innate immune system represents a clinically attractive target in CF to improve lung function. NETs and IL-1β represent two important components of CF lung inflammation. Degrading or inhibiting the formation or activities of NETs, IL-1β, or both, are expected to benefit PwCF. Extracellular DNA is hydrolyzed by the human endonuclease, deoxyribonuclease I (DNase I). Recombinant DNase targeting NETs significantly reduces airway levels of extracellular DNA and improves lung function in PwCF (175). DNase I is the only mucolytic agent with proven efficacy in CF and remains recommended to be given, even in the era of effective CFTR modulators (175). Currently, there are no therapies approved for PwCF that specifically target NET formation (40).

Targeting IL-1β therapeutically became first feasible by the introduction of anakinra, a recombinant and non-glycosylated form of the naturally occurring IL-1 receptor antagonist (IL-1Ra) (176). While anakinra has not been approved for PwCF to date, results of animal models and preclinical studies suggest it could represent a beneficial anti-inflammatory therapy in CF with no or minimal side effects (177). Canakinumab (ACZ885) is a human anti-IL-1β monoclonal neutralizing antibody (Novartis) that was approved by the Food and Drug Administration in the U.S.A. in 2009 for the treatment of familial cold auto-inflammatory syndrome and Muckle-Wells syndrome (178) but it has not been approved for PwCF.

Accumulating data suggest that CFTR correctors have beneficial effects on chronic lung inflammation in CF but failed to resolve it entirely (167, 179, 180). Trikafta therapy significantly increases CFTR protein expression and reduces ATP/P2X7R-induced NLRP3 inflammasome activation, reducing airway inflammation in PwCF (167).

As theoretical, additional possibilities, activation of the inflammasomes, caspase-1 or NET formation could be inhibited by specific inhibitors. Whether these, other, anti-inflammatory approaches, or their combinations with each other or CFTR correctors, will be clinically feasible in CF, will be decided in the future.

## 6 Conclusions

Altogether, cells in the CF airway demonstrate dysregulated NLRP3 inflammasome signaling characterized by enhanced IL-1β secretion. A characteristic of CF lung disease is the infiltration of neutrophils releasing NETs that could be potentially enhanced by IL-1β. Microbial infections in the CF lung induce NET release which induces more NLRP3 activation in macrophages and keeps feeding chronic inflammation. We acknowledge that focusing on NETs and IL-1β in this manuscript is somewhat subjective and several other mechanisms of the innate immune system take place in the lungs of PwCF that could drive lung disease. The data summarized in this review article propose, however, that a self-perpetuating, positive feedback loop consisting of IL-1β and NETs represent a significant driving force behind CF lung disease progression (**Figure 1**). While both mechanisms are complex and relevant to fight infections in healthy individuals, data accumulated thus far mainly suggests their overall pathologic, not beneficial, role in CF. Therefore, understanding the pathways associated with inflammasome activation and NET formation could aid in developing new therapeutics for PwCF to prevent or reduce chronic lung disease.
